# Occurrence of Pesticide Residues and Acute, Chronic, and Cumulative Dietary Risk Assessment in Mango (*Mangifera indica* L.) from Lower Northern Thailand

**DOI:** 10.3390/foods15162856

**Published:** 2026-08-16

**Authors:** Kanlayanee Boonthawee, Phannika Tongchai, Pichamon Yana, Udomsap Jaitham, Peerapong Jeeno, Nid Lungmala, Sumed Yadoung, Khanchai Danmek, Panamas Treewannakul, Yuichiro Amekawa, Surat Hongsibsong

**Affiliations:** 1 Environmental, Occupational Health Sciences and NCD Center of Excellence, Research Institute for Health Sciences, Chiang Mai University, Chiang Mai 50200, Thailand; kanlayanee_b@cmu.ac.th (K.B.); phannika.t@cmu.ac.th (P.T.); pichamon.y@cmu.ac.th (P.Y.); udomsap_j@cmu.ac.th (U.J.); peerapong_jeen@cmu.ac.th (P.J.); nid_lungmala@cmu.ac.th (N.L.); sumed.yadoung@cmu.ac.th (S.Y.); 2 School of Health Sciences Research, Research Institute for Health Sciences, Chiang Mai University, Chiang Mai 50200, Thailand; 3 Office of Research Administration, Chiang Mai University, Chiang Mai 50200, Thailand; 4 School of Agriculture and Natural Resources, University of Phayao, Phayao 56000, Thailand; khanchai.da@up.ac.th; 5 Faculty of Agriculture, Kasetsart University, Bangkok 20900, Thailand; panamas.t@ku.th; 6 College of International Relations, Ritsumeikan University, Kyoto 603-8577, Japan

**Keywords:** mango, pesticide residues, QuEChERS, GC–MS/MS, dietary risk assessment, hazard index

## Abstract

Mango (*Mangifera indica* L.), particularly the ‘Nam Dok Mai’ cultivar, is an economically important fruit crop in Thailand and is widely produced in the Lower Northern Region for domestic consumption and export. However, pesticide residues in mango remain a major food safety concern because of their potential effects on consumer health and compliance with national and international maximum residue limits (MRLs). This study investigated pesticide residue contamination and assessed the dietary health risks associated with mango consumption among different age groups. A total of 255 composite mango samples were collected from production areas in Phichit, Phitsanulok, and Phetchabun provinces, Thailand. Pesticide residues were analyzed using a validated modified QuEChERS extraction method combined with gas chromatography–tandem mass spectrometry (GC–MS/MS). Multiple pesticide residues were frequently detected in the samples analyzed. Of the detected residues, 22 pesticides exceeded Thai MRL standards and 14 exceeded Codex Alimentarius MRLs. Dietary exposure was evaluated using acute and chronic hazard quotients (HQa and HQc) and cumulative hazard indices (HIa and HIc). The results showed that acute exposure to several insecticides, particularly carbofuran, lambda-cyhalothrin, and fenpropathrin, exceeded acceptable risk thresholds in all age groups. At the pesticide-class level, cumulative acute exposure to organophosphates, carbamates, and pyrethroids showed HIa values greater than 1 in several age groups, suggesting potential short-term health concerns, especially among children. In contrast, chronic exposure to individual pesticides and cumulative pesticide groups remained below the level of concern for all age groups, with HQc and HIc values below 1. These findings indicate that although long-term dietary risk from mango consumption is within acceptable levels, acute exposure to certain pesticide residues may pose non-negligible health risks. Continuous residue monitoring, stricter enforcement of good agricultural practices, and evidence-based risk management are essential to ensure mango safety and protect consumers.

## 1. Introduction

Pesticide residues in food commodities remain a significant global concern for food safety, public health, and international trade. Pesticides are widely used in modern agricultural production to control insects, weeds, and plant diseases, thereby improving crop yield and market value. However, inappropriate pesticide use, including excessive application, improper mixing, repeated spraying, and failure to comply with recommended pre-harvest intervals, can result in the accumulation of pesticide residues in agricultural products. When pesticide residue levels exceed established maximum residue limits (MRLs), they may pose potential health risks to consumers and result in non-compliance with national and international food safety standards [1,2].

Fruits are among the agricultural commodities most frequently associated with pesticide residue concerns because they are often treated repeatedly during cultivation to maintain their appearance and quality. In addition, many fruits are consumed fresh or with minimal processing, which may increase the likelihood of dietary exposure to residual pesticides. Previous monitoring studies have reported the occurrence of multiple pesticide residues in fruit samples, including organophosphates, carbamates, pyrethroids, organochlorines, and herbicides [1,3]. The co-occurrence of several pesticide residues in a single commodity is particularly concerning because consumers may be exposed not only to individual compounds but also to pesticide mixtures with similar toxicological effects, potentially resulting in cumulative exposure and additive health risks [4,5].

Mango (*Mangifera indica* L.) is one of Thailand’s most economically important fruit crops. The ‘Nam Dok Mai’ cultivar is particularly valued for its attractive appearance, sweet flavor, and favorable texture, making it important for both domestic consumption and export markets. Mangoes from Thailand are supplied to high-value markets where strict food safety regulations and pesticide residue standards are enforced. Therefore, pesticide residues in mango may affect consumer confidence, trade acceptance, and compliance with food safety requirements. In the Lower Northern Region of Thailand, including Phichit, Phitsanulok, and Phetchabun provinces, mango production is an important agricultural activity. These areas are characterized by intensive fruit cultivation, and farmers commonly rely on synthetic pesticides to manage insect pests and maintain fruit quality for commercial distribution [1,2]. The toxicological significance of pesticide residues depends on the type of compound, residue concentration, consumption amount, and consumer characteristics such as age and body weight. Organophosphate and carbamate insecticides are of particular concern because they inhibit acetylcholinesterase activity and may cause acute neurotoxic effects [3,4,5]. Pyrethroid insecticides act mainly on voltage-gated sodium channels and may also contribute to neurotoxic outcomes at elevated exposure levels [6]. Organochlorine pesticides, although restricted or banned in many countries, may persist in the environment and remain relevant in residue monitoring [5,7]. Herbicides such as atrazine may also be of concern because of their potential endocrine-related effects [8]. Therefore, dietary exposure assessment is necessary to characterize both short-term and long-term health risks associated with pesticide residues in mango [9,10,11].

Dietary risk assessment commonly uses hazard quotient and hazard index approaches to evaluate potential health risks from pesticide intake. Acute risk assessment estimates short-term exposure using the highest detected residue and large portion consumption of the commodity, whereas chronic risk assessment estimates long-term exposure using mean residue concentrations and average food consumption [12,13]. In addition to individual pesticide assessment, cumulative risk assessment is important for pesticide groups that share similar toxicological profiles or modes of action. This approach provides a more realistic evaluation of consumer exposure, particularly when multiple residues are detected in the same food commodity [4,5,14].

Reliable assessment of pesticide residues requires sensitive and selective analytical methods. The QuEChERS extraction method combined with gas chromatography–tandem mass spectrometry (GC–MS/MS) has been widely applied for multi-residue analysis in fruits and vegetables because it is rapid, efficient, and suitable for detecting trace levels of multiple pesticide compounds in complex food matrices [11,12]. Although multi-residue pesticide monitoring and dietary risk assessment in mango have been reported previously, evidence remains limited for ‘Nam Dok Mai’ mangoes produced in the Lower Northern Region of Thailand. In particular, data integrating pesticide-residue occurrence, compliance with Thai and Codex MRLs, and age-specific cumulative dietary risk assessment using Thai exposure parameters remain limited. Such information is needed to support risk-based monitoring and pesticide-management strategies in regional mango supply chains.

The international relevance of pesticide-residue surveillance in Thai mangoes extends beyond local food safety because residue compliance can influence market access in export supply chains. Comparison with Codex MRLs provides a harmonized and internationally recognized reference for evaluating pesticide-residue compliance in trade, while region-specific occurrence data can support targeted monitoring and pesticide-management strategies at the production level.

The present study employed a targeted, rather than exhaustive, pesticide-residue monitoring approach. The 44 target pesticides were selected based on three main considerations: their analytical suitability for multi-residue determination using the established EN QuEChERS–GC–MS/MS method, including the availability of reference standards and appropriate mass transitions; their toxicological relevance to dietary exposure assessment; and their relevance to pesticide-residue surveillance in fruit commodities [13,14]. The target panel covered major insecticide classes, including organophosphates, carbamates, and pyrethroids, as well as selected organochlorine pesticides, amitraz, and atrazine.

Selected persistent organochlorine pesticides, including aldrin, endosulfan, and DDT-o,p′, were included for surveillance purposes because such residues may remain detectable in agricultural environments despite regulatory restrictions or prohibitions [15,16]. The analytical panel was not intended to encompass all pesticides registered for mango production in Thailand, nor to determine pesticide-use authorization at the farm level. Registration status and authorized uses may vary according to pesticide formulation, crop, target pest, and regulatory period. Therefore, the detection of a residue in mango samples should not, by itself, be interpreted as evidence of unauthorized or illegal pesticide use.

Therefore, this study aimed to determine the occurrence and concentration levels of 44 target pesticide residues in ‘Nam Dok Mai’ mango samples collected from production areas in Phichit, Phitsanulok, and Phetchabun provinces, Thailand. The detected residues were compared with Thai and Codex MRLs to evaluate regulatory compliance. In addition, individual acute and chronic dietary exposure assessments, together with cumulative risk assessments for selected pesticide groups, were conducted across different age groups using hazard quotient (HQ) and hazard index (HI) approaches. This study provides region- and cultivar-specific residue data and age-specific cumulative dietary risk information based on Thai exposure assumptions. These findings provide baseline evidence for risk-based pesticide monitoring, improved agricultural practices, and food-safety management in Thai mango supply chains serving domestic and export markets.

## 2. Materials and Methods

### 2.1. Chemicals and Reagents

The target analytes comprised 44 pesticide compounds, including organophosphates, pyrethroids, organochlorines, carbamates, and herbicides. Analytical-grade pesticide standards with purity of ≥99% were obtained from Dr. Ehrenstorfer GmbH (Augsburg, Germany). Individual stock standard solutions were prepared at a concentration of 1000 µg/mL in acetonitrile and stored under appropriate conditions until analysis. Working standard solutions were prepared by serial dilution of the stock solutions with acetonitrile. Acetonitrile, ethyl acetate, and ultrapure water were used for standard preparation and sample extraction. Acetonitrile and ethyl acetate were of HPLC grade and were purchased from J.T. Baker, Avantor Performance Materials, LLC (Radnor Township, PA, USA). The QuEChERS extraction salts consisted of magnesium sulfate (MgSO_4_), sodium chloride (NaCl), trisodium citrate dihydrate (Na_3_C_6_H_5_O_7_·2H_2_O), and disodium hydrogen citrate sesquihydrate (Na_2_HC_6_H_5_O_7_·1.5H_2_O). High-purity helium (99.999%) was used as the carrier gas, while high-purity nitrogen (99.999%) was used as the collision and quench gas for gas chromatography–tandem mass spectrometry (GC–MS/MS) analysis was performed using an Agilent 8890 Gas Chromatograph, equipped with a 7693A autosampler and a 7000E triple quadrupole mass spectrometer, operated through Mass Hunter Software Quantitative Analysis version B.12.1.938.3 installed on a dedicated workstation for data acquisition and processing (Agilent Technologies, Inc., Santa Clara, CA, USA).

### 2.2. Sample Collection

**Figure 1:**
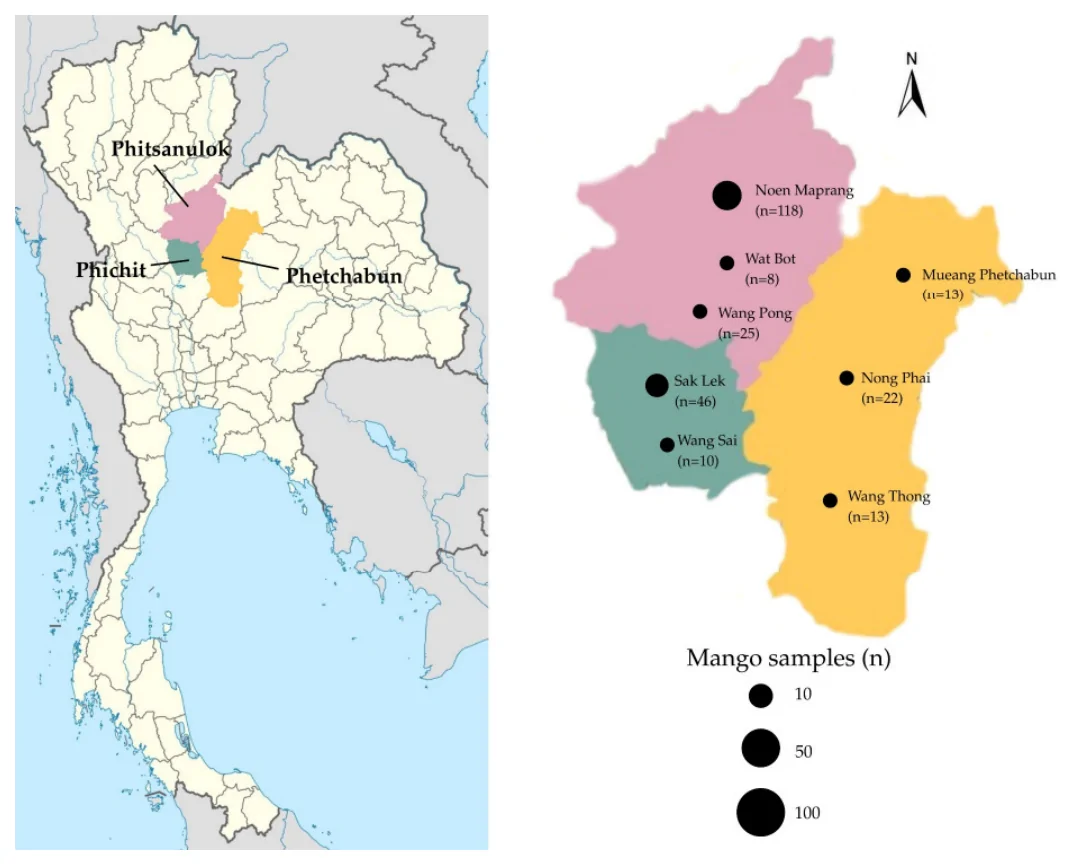
Field survey provinces, Thailand (Pink: Phitsanulok; Green: Phichit; Yellow: Phetchabun).

Mango samples of the ‘Nam Dok Mai’ cultivar were collected from mango-producing areas in the Lower Northern Region of Thailand, including Phichit, Phitsanulok, and Phetchabun provinces, between April and July 2024 (Figure 1). The sampling areas were selected from representative mango cultivation districts within the three study provinces. A total of 255 composite mango samples were collected, comprising 56 samples from Phichit, 139 samples from Phitsanulok, and 60 samples from Phetchabun.

At the district level, samples were collected from Sak Lek (*n* = 46) and Wang Sai (*n* = 10) districts in Phichit; Noen Maprang (*n* = 118), Wat Bot (*n* = 8), and Wang Thong (*n* = 13) districts in Phitsanulok; and Wang Pong (*n* = 25), Nong Phai (*n* = 22), and Mueang Phetchabun (*n* = 13) districts in Phetchabun (Figure 1). As individual orchard coordinates were not recorded, the spatial distribution of samples is presented at the district level.

Each orchard was treated as a cluster sampling unit. For each sampled orchard, the cultivation area was divided into four sections, and one mango tree was selected from each section. One mango fruit at approximately 70–80% maturity was collected from a randomly selected position on each selected tree. The four fruits collected from each orchard were transported to the research team and pooled to form one composite sample weighing approximately 1 kg.

After collection, all composite samples were transported to the laboratory under appropriate conditions. Each sample was thoroughly homogenized before extraction, transferred to clean containers, and stored at −20 °C until pesticide residue analysis.

### 2.3. Sample Preparation and Extraction

Mango samples were prepared without prior washing by removing the seeds, and both the pulp and peel were chopped into small pieces. Pesticide residues in the mango samples (5 g) were extracted using the EN QuEChERS method [16]. Weighing was performed into 50 mL centrifuge tubes. For method validation, samples were spiked with pesticide standard mixtures to obtain final concentrations of 10, 100, and 500 µg/kg. A non-fortified sample was prepared as a blank matrix for matrix-matched calibration. For extraction, 15 mL of acetonitrile was added to each tube, followed by thorough mixing. The tubes were sealed and shaken for 5 min using a SPEX 2000 Geno Grinder (Digital Multi-Tube Vortex Mixer, Model VXMTDG, OHAUS, Parsippany, NJ, USA) at 2500 rpm. Subsequently, a QuEChERS extraction salt mixture (4 g MgSO_4_, 1 g NaCl, 1 g Na_3_C_6_H_5_O_7_·2H_2_O, and 0.5 g Na_2_HC_6_H_5_O_7_·1.5H_2_O) was added. The samples were immediately shaken again for 5 min under the same conditions and then centrifuged at 2000 rpm for 5 min. An aliquot of the supernatant was transferred and evaporated to dryness at 40 °C using a gentle stream of nitrogen. The residue was reconstituted in 2 mL of ethyl acetate and subsequently filtered through a 0.45 µm syringe filter into GC vials. Finally, 1 µL of the extract was injected into a gas chromatography–tandem mass spectrometry (GC–MS/MS) system for analysis.

### 2.4. GC–MS/MS Analytical Conditions

Pesticide residue analysis was performed using an Agilent 8890 Gas Chromatograph coupled with an Agilent 7000E Triple Quadrupole Mass Spectrometer (Agilent Technologies, Santa Clara, CA, USA). Chromatographic separation was achieved on an HP-5ms Ultra Inert capillary column (30 m × 0.25 mm i.d., 0.25 µm film thickness) (Agilent Technologies, Santa Clara, CA, USA). Helium with a purity of 99.999% was used as the carrier gas at a constant flow rate of 1.0 mL/min. An aliquot of 1 µL of the final extract was injected in spitless mode, and the inlet temperature was maintained at 250 °C. The oven temperature program was set as follows: initial temperature of 70 °C, held for 2 min; increased to 150 °C at 25 °C/min; increased to 200 °C at 3 °C/min; and finally increased to 280 °C at 8 °C/min and held for 10 min.

The mass spectrometer was operated in electron ionization (EI) mode at 70 eV. Detection and quantification were performed in multiple reaction monitoring (MRM) mode to provide high sensitivity and selectivity for the target pesticides. High-purity nitrogen (99.999%) was used as both the collision and quench gas. The transfer line, ion source, and quadrupole temperatures were maintained at 280 °C, 230 °C, and 150 °C, respectively. Data acquisition and processing were performed using Mass Hunter Quantitative Analysis software, version B.12.1.938.3.

### 2.5. Acute Risk Assessment

The acute health risk to consumers (HQa) was determined from the estimated short-term dietary intake (ESTI) in relation to the acute reference dose (ARfD). The ESTI for each pesticide in mango was calculated using Equation (1) in accordance with the JMPR guidelines [9,11].

(1) $$ESTI=\frac{HR\times LP}{BW}$$

The HQa of a pesticide was estimated using the following Equation (2).

(2) $$HQa\;(\%)=\frac{ESTI}{ARfD}\times 100$$

where ESTI estimates the short-term intake of each pesticide by consumption, µg/kg bw, HR is the highest measured residue concentration of a pesticide in available samples, µg/kg, LP is the large-portion consumption (P97.5), kg/d, BW is the body weight of adults and children, kg, ARfD is the acute reference dose (µg/kg BW). Acute risk was considered acceptable when HQa < 100% and unacceptable when HQa ≥ 100%. In this study, the combination of HR and LP was used to represent a worst-case high-exposure scenario in line with JMPR acute dietary risk assessment practice. The body weight, large-portion consumption, and average daily consumption values used in the acute risk assessment for different age groups are presented in Table 1.

### 2.6. Chronic Risk Assessment

The long-term (chronic) dietary risk (HQc) was assessed by relating the estimated daily intake (EDI) of each pesticide to its acceptable daily intake (ADI). The EDI for pesticides in mango was obtained using Equation (3).

(3) $$EDI=\sum \frac{C\times F}{BW}$$

The HQc of pesticide was estimated using the following Equation (4).

(4) $$HQc\;(\%)=\frac{EDI}{ADI}\times 100$$

where EDI is the estimated daily intake of a pesticide through mango consumption (µg/kg bw/day), C is the average residue data of each pesticide (µg/kg), F is the average daily consumption (kg/day), BW is the average body weight (kg), and ADI is the acceptable daily intake (µg/kg bw/day). Not detected (ND), Were assigned a value of zero. Mean residue concentrations were calculated using all 255 mango samples; therefore, EDI and HQc represent lower-bound estimates of chronic dietary exposure.

Chronic risk was considered acceptable when HQc was <100% and unacceptable when HQc was ≥100%.

### 2.7. Hazard Index (HI)

The cumulative risk hazard index (HI) related to multiple pesticide consumption was the sum of HQn using the following Equation (5).

(5) $$HI=\sum_{n=1}^{i}HQn$$

When HI is ≥1, it indicates that pesticide residue could pose health risk to consumers.

## 3. Results and Discussion

### 3.1. Method Validation Parameters for the Determination of Pesticide Residues

**Figure 2:**
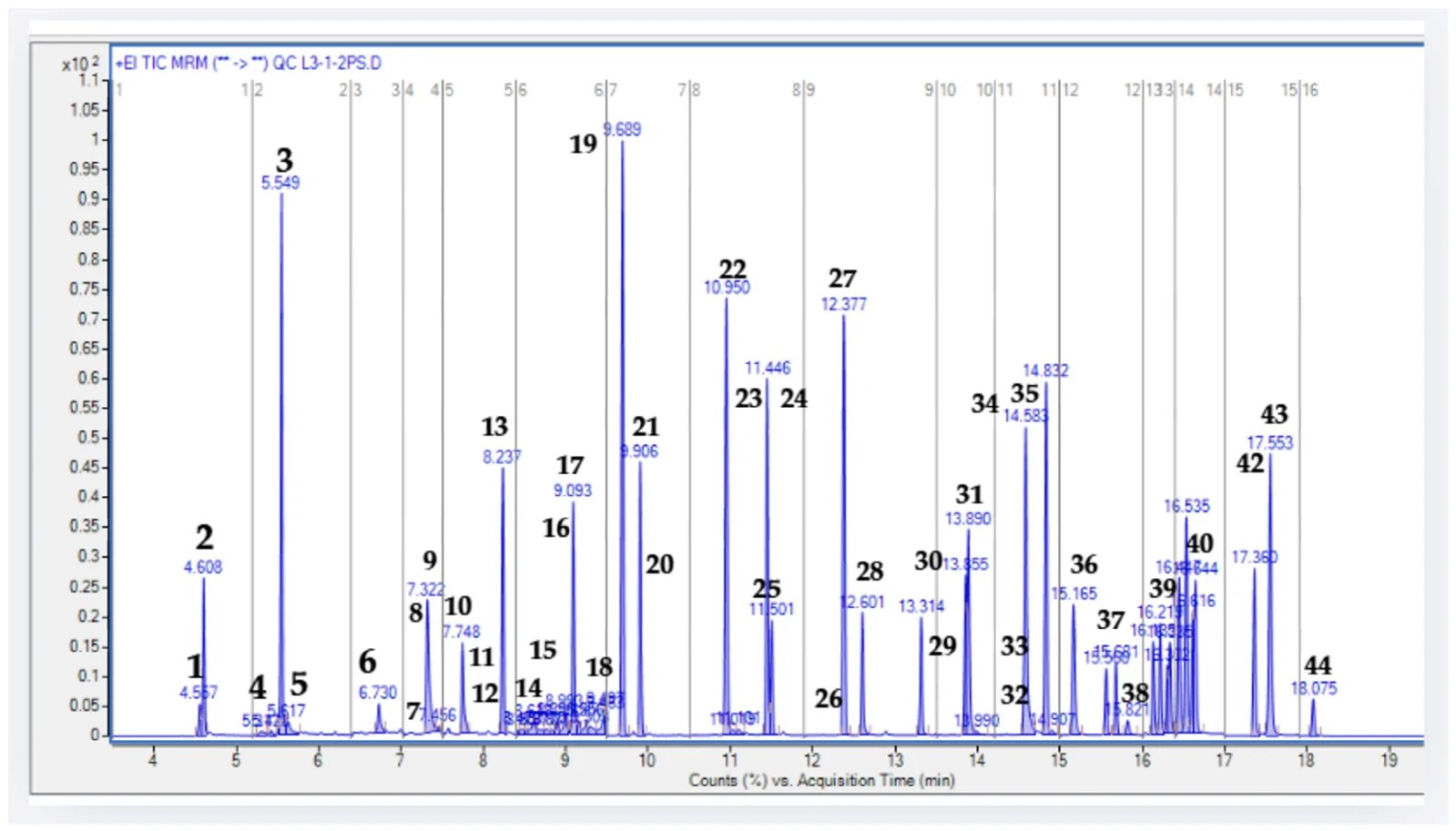
Representative total ion chromatogram (TIC) in multiple reaction monitoring (MRM) mode of a 44-pesticide standard mixture. The numbered peaks were identified as follows: 1, methamidophos (RT 4.557 min); 2, dichlorvos (RT 4.608 min); 3, E-mevinphos (RT 5.549 min); 4, acephate (RT 5.621 min); 5, oxamyl (RT 5.686 min); 6, omethoate (RT 6.731 min); 7, fenobucarb (RT 6.996 min); 8, dicrotofos (RT 7.324 min); 9, monocrotophos (RT 7.325 min); 10, dimethoate (RT 7.749 min); 11, atrazine (RT 7.769 min); 12, carbofuran (RT 7.792 min); 13, diazinon (RT 8.238 min); 14, pirimicarb (RT 8.992 min); 15, pirimiphos-methyl (RT 9.092 min); 16, fenitrothion (RT 9.094 min); 17, parathion-methyl (RT 9.094 min); 18, carbaryl (RT 9.257 min); 19, malathion (RT 9.690 min); 20, aldrin (RT 9.823 min); 21, chlorpyrifos (RT 9.906 min); 22, methidathion (RT 10.950 min); 23, prothiofos (RT 11.446 min); 24, endosulfan (RT 11.447 min); 25, profenofos (RT 11.500 min); 26, o,p′-DDT (RT 12.311 min); 27, ethion (RT 12.378 min); 28, triazophos (RT 12.602 min); 29, carbosulfan (RT 13.667 min); 30, phosmet (RT 13.855 min); 31, EPN (RT 13.891 min); 32, amitraz (RT 14.305 min); 33, azinphos-methyl (RT 14.565 min); 34, fenpropathrin (RT 14.583 min); 35, lambda-cyhalothrin (RT 14.584 min); 36, azinphos-ethyl (RT 15.166 min); 37, cis-permethrin (RT 15.681 min); 38, coumaphos (RT 15.822 min); 39, cyfluthrin I (RT 16.220 min); 40, cypermethrin I (RT 16.645 min); 41, fenvalerate I (RT 17.553 min); 42, esfenvalerate (RT 17.553 min); 43, tau-fluvalinate II (RT 17.554 min); and 44, deltamethrin (RT 18.075 min). Note: ** denotes all monitored precursor or product ions in the MRM transitions.

**Figure 3:**
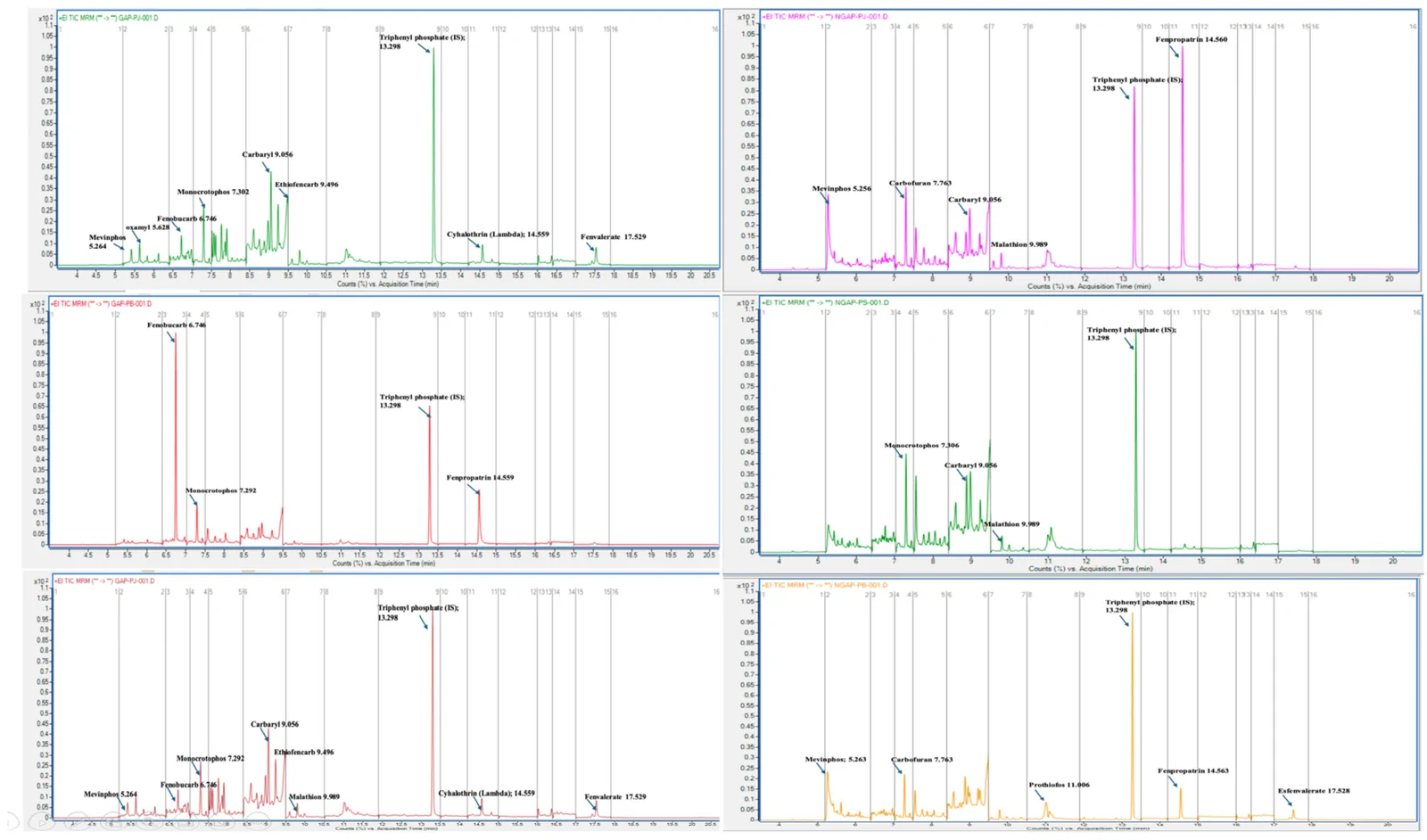
Representative GC–MS/MS TIC-MRM chromatograms from selected mango samples. Selected pesticide residues are labeled with their retention times (min), and triphenyl phosphate was used as the internal standard (IS; RT = 13.298 min). Note: ** denotes all monitored precursor or product ions in the MRM transitions.

The validation results confirmed that the developed GC–MS/MS method was suitable for the determination of 44 pesticide residues in mango matrices. Representative GC–MS/MS TIC-MRM chromatograms of the quality control sample and selected mango samples are presented in Figure 2 and Figure 3.

The method showed good linearity for most target analytes, with correlation coefficients (r^2^) ranging from 0.9563 to 0.9998. Most compounds exhibited r^2^ values greater than 0.99, indicating reliable calibration performance across the evaluated concentration range.

The method validation parameters, including mass transitions, linearity, limits of detection (LODs), limits of quantitation (LOQs), precision, and recovery, are summarized in Table 2. Method precision was evaluated as the relative standard deviation (%RSD) at a fortification level of 100 µg/kg (*n* = 10). The %RSD values ranged from 1.00% to 15.58%, with most analytes showing values below 10%, indicating good repeatability. All target compounds met the acceptable precision criterion of ≤20%, confirming satisfactory method precision. Method accuracy was assessed through recovery experiments at three fortification levels: 10, 100, and 500 µg/kg. The recoveries ranged from 78.48% to 121.39% at 10 µg/kg, 74.04% to 103.23% at 100 µg/kg, and 85.21% to 106.48% at 500 µg/kg. Most analytes were within the commonly accepted recovery range of 70–120%, confirming the accuracy of the method. Overall, the validation results demonstrated that the modified QuEChERS extraction combined with GC–MS/MS analysis provides a reliable, precise, accurate, and sensitive method for multi-residue pesticide determination in mango matrices.

### 3.2. Occurrence of Pesticide Residues in Mango Samples

A total of 255 mango samples were analyzed for 44 pesticide residues belonging to five major classes: organophosphates (24 compounds), carbamates and related insecticides (7 compounds), pyrethroids (9 compounds), organochlorines (4 compounds), and one herbicide, atrazine. Table 3. summarizes the detection frequencies, residue concentrations, and corresponding Thai and Codex maximum residue limits (MRLs).

Pesticide residues were frequently detected in mango samples. Chlorpyrifos was the most frequently detected compound, occurring in 134 samples (52.5%) with a mean concentration of 3.8 µg/kg. This residue level did not exceed either the Thai or Codex MRLs. Esfenvalerate was the most frequently detected pyrethroid, found in 131 samples (51.4%) with a mean concentration of 31.9 µg/kg, remaining below both Thai and Codex MRLs. Amitraz was detected in 117 samples (45.9%) with a mean concentration of 61.4 µg/kg. This concentration was below the Thai MRL of 2000 µg/kg but exceeded the Codex MRL of 10 µg/kg by approximately 6.14-fold. Carbofuran, a highly toxic carbamate insecticide, was detected in 96 samples (37.6%), indicating its continued use and relevance for dietary risk assessment. The high detection frequencies of chlorpyrifos, esfenvalerate, amitraz, and carbofuran may reflect the intensive use of insecticides in mango production, particularly organophosphates, pyrethroids, and carbamates. This pattern is consistent with national reports describing the widespread application of pesticides in Thai agriculture, especially organophosphate and pyrethroid compounds [17]. Similar findings have also been reported in international fruit-monitoring studies, where organophosphate and pyrethroid residues are commonly detected because of their broad-spectrum activity and extensive use in pest management programs [18,19].

Based on the comparison with the corresponding MRLs, mean residue concentrations of 20 out of 44 pesticides were found to be higher than the Thai regulatory limits. Most of these compounds were subject to the default MRL of 10 µg/kg, as specific MRLs for mango have not been established in Thailand. For Codex standards, 14 pesticides had mean concentrations exceeding the applicable MRLs, mainly among compounds assigned the default Codex MRL of 10 µg/kg for non-registered uses. In addition, 10 compounds were classified as “not established” (NE) under Codex, indicating the absence of pesticide–commodity-specific Codex MRLs for mango.

Within the organophosphate group, acephate, monocrotophos, and several related compounds had mean concentrations higher than the default Thai MRL of 10 µg/kg. Acephate showed a mean concentration of 106.2 µg/kg, reflecting a relatively elevated residue level compared with the default limit. In the carbamate group, oxamyl (98.3 µg/kg), fenobucarb (93.9 µg/kg), and carbofuran also showed mean concentrations higher than the Thai MRLs. These results indicate that these compounds should be prioritized for continued monitoring and that improved adherence to recommended pesticide application practices may be needed in mango orchards. Pyrethroids generally showed higher residue concentrations than other pesticide classes. Lambda-cyhalothrin and fenpropathrin were detected at mean concentrations of 1009.4 and 1222.4 µg/kg, respectively, which were above their corresponding Thai MRLs. These findings suggest that these compounds should be prioritized for further monitoring and improved pesticide management in mango production. Other pyrethroids, including permethrin and cypermethrin, also exceeded Thai MRLs, although to a lesser extent. For organochlorine pesticides, aldrin, endosulfan, and DDT-o,p′ were detected at relatively low mean concentrations and generally remained below both Thai and Codex MRLs. In contrast, amitraz exceeded only the Thai MRL while remaining compliant with the Codex limit. The herbicide atrazine was detected sporadically and consistently remained below the applicable regulatory limits.

Overall, the results indicate that pesticide residues from multiple insecticide classes were commonly detected in mango samples from the study areas. The presence of compounds exceeding Thai or Codex MRLs highlights the need for continued residue surveillance, improved pesticide management, and stricter compliance with good agricultural practices in mango production. Moreover, the detection of several organophosphate compounds classified as NE under Codex, including mevinphos, omethoate, and monocrotophos, may complicate compliance assessment for international trade and underscores the need for further consideration of appropriate Codex MRLs for mango [18,19].

### 3.3. Acute Intake Risk

Acute dietary risk from mango consumption was evaluated for all pesticides with an established acute reference dose (ARfD) by comparing the estimated short-term intake (ESTI) with the corresponding ARfD across six age groups (3.0–5.9, 6.0–12.9, 13.0–17.9, 18.0–34.9, 35.0–64.9, ≥65 years), as summarized in Table 4. For several pesticides, including dicrotophos, ethion, azinphos-ethyl and DDT o,p′, no ARfD has yet been established by major regulatory bodies, and therefore quantitative acute risk characterization could not be performed. Within the organophosphate group, none of the pesticides with defined ARfD values showed ESTI exceeding ARfD in any age group, and all corresponding HQa values remained below 100% [10]. This indicates that acute exposure to organophosphate residues through mango consumption under the studied conditions is unlikely to pose an immediate health risk, which is consistent with previous multi-residue studies in fruits that also reported HQa values for organophosphates below the acute concern level [20].

In contrast, several carbamate pesticides contributed substantially to acute risk. Carbofuran showed the highest concern, with a maximum residue level of 362.728 µg/kg and an ARfD of 1 µg/kg BW. The ESTI values for carbofuran ranged from 3.317 to 5.362 µg/kg BW across the age groups, corresponding to approximately 3.3–5.5-fold exceedance of the ARfD. The highest ESTI was observed in children aged 6–12.9 years (5.542 µg/kg BW), reflecting their higher fruit intake per kilogram body weight compared with adults. Consequently, HQa (%) for carbofuran exceeded 100% in all age groups, with the greatest values in “school aged children”, indicating a potential acute health concern for this vulnerable population. These results are particularly concerning because carbofuran is recognised as a highly toxic carbamate insecticide that has been banned or severely restricted in many countries due to its acute neurotoxicity and risk to vulnerable populations. Similar findings have been reported in other dietary risk assessments, where carbofuran and related carbamate insecticides made a disproportionate contribution to acute risk indicators despite relatively low detection frequencies [21].

Among pyrethroid insecticides, lambda-cyhalothrin and fenpropathrin were identified as the main drivers of acute risk. The maximum residues of lambda-cyhalothrin and fenpropathrin were 10,272.443 and 11,439.066 µg/kg, with ARfD values of 20 and 30 µg/kg bw, respectively. The resulting ESTI values exceeded the ARfD by approximately 4.7–7.6 times for lambda-cyhalothrin and 3.5–5.6 times for fenpropathrin, depending on the age group, with the highest exceedances again occurring in children aged 6–12.9 years. These exceedances are reflected in HQa (%) values greater than 100% for all age groups, indicating that these two pyrethroids are major contributors to the acute dietary risk associated with mango consumption in the present study. These results indicate that, under the conditions of this study, organochlorine and herbicide residues in mango do not pose an appreciable acute health risk to consumers.

Overall, while many pesticides showed HQa values well below 100%, the exceedances observed for carbofuran, lambda-cyhalothrin and fenpropathrin suggest that acute exposure to these compounds may not be negligible. These results underline the importance of enforcing good agricultural practices, reviewing the use of highly toxic carbamate and pyrethroid insecticides in mango cultivation, and implementing targeted risk-management measures to reduce acute dietary exposure in sensitive population groups [8].

### 3.4. Chronic Intake Risk

Chronic dietary risk was evaluated using HQc to estimate long-term dietary exposure to pesticide residues through mango consumption (Table 5). For all pesticides included in this study, the EDI values were lower than their respective ADI in every age group, resulting in HQc values below 100% for all compounds. These findings are consistent with international and national risk assessment guidelines, which consider HQ or %ADI below 100% as indicative of an acceptable chronic risk level for consumers. For example, chronic dietary risk assessments conducted by the Thai Department of Agriculture and related studies on fruits have similarly reported HQ values well below 1 (or 100%) and concluded that pesticide exposure via consumption of these commodities is within safe limits [22].

These findings are consistent across different pesticide classes, including organophosphates, carbamates, pyrethroids, organochlorines and the herbicide atrazine, suggesting that long-term exposure to individual pesticide residues from mango consumption alone is unlikely to pose appreciable chronic health risks for either children or adults.

However, it should be noted that this chronic risk assessment considered only mango as a single dietary source; consumers may also be exposed to pesticide residues from other foods, which can contribute to their overall long-term dietary exposure. Therefore, while our results indicate that, under the current residue levels and consumption scenarios, the chronic intake of pesticide residues through mango is within the established safety margins, although continued monitoring and adherence to good agricultural practices remain essential to ensure sustained consumer protection.

### 3.5. The Cumulative Exposure Risk Assessment of Mango

The hazard index (HI) approach was applied to evaluate the cumulative non-carcinogenic risk from exposure to multiple pesticide residues through mango consumption. This approach is suitable for chemicals that share similar toxicological effects or modes of action and has been widely used for cumulative dietary risk assessment of pesticide mixtures in foods [23,24].

**Figure 4:**
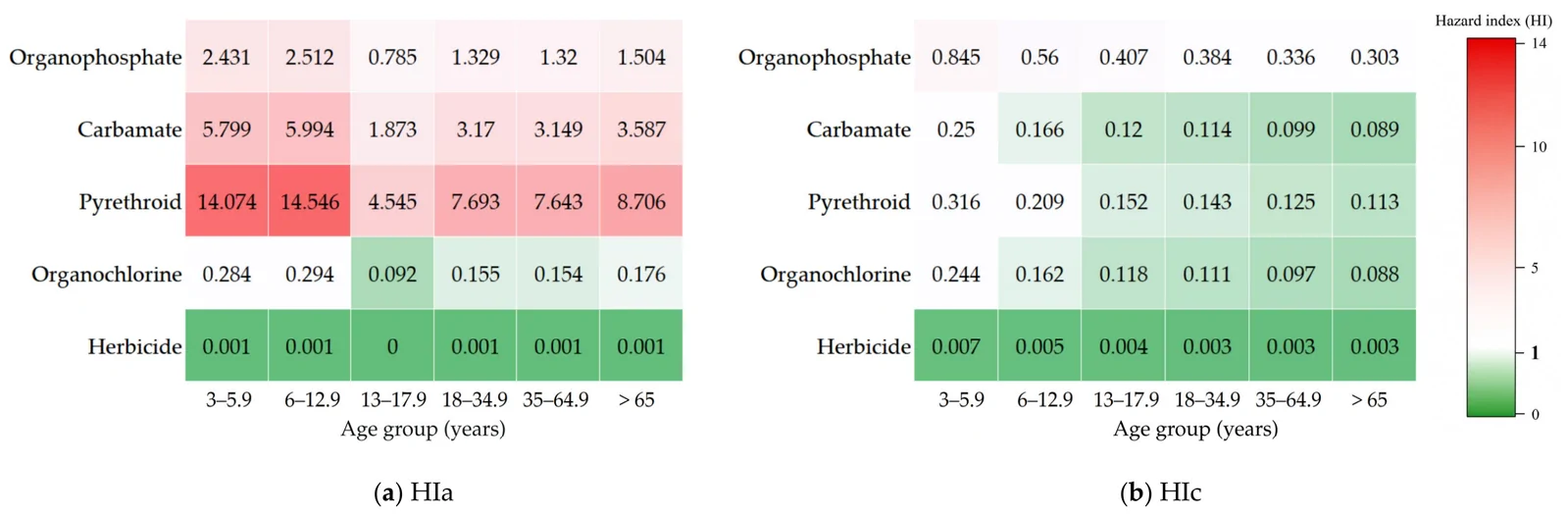
Heatmap of cumulative hazard indices (HI) for pesticide groups across age groups: (**a**) acute cumulative hazard index (HIa); (**b**) chronic cumulative hazard index (HIc). Both panels share the same colour scale, shown by the vertical colour bar, which maps HI values from 0 (green) to 14 (dark red).

Based on acute cumulative risk visualized in Figure 4a, a distinct pattern of elevated risk is clearly observable in the red-shaded regions, where HIa values for several pesticide classes exceeded the acceptable threshold of 1. Specifically, organophosphates exceeded 1 in almost all age groups, ranging from 1.320 to 2.512, except in the 13–17.9 age group, which is represented by a safe green hue (HIa = 0.785), due to higher body weight and relatively lower effective mango consumption per kilogram body weight. These findings indicate that the combined acute exposure to organophosphate residues may exceed the acceptable threshold in several age groups, particularly among younger children.

For carbamates, the HIa values ranged from 1.873 to 5.994, indicating a higher level of acute cumulative risk, likely driven by highly potent compounds such as carbofuran. Pyrethroids demonstrated the greatest acute cumulative risk, appearing as the most intensely colored red blocks on the heatmap, with HIa values ranging from 4.543 to 14.546. This suggests that the pesticide group contributed most strongly to acute exposure concerns in the studied mango samples [6,20]. In contrast, the HIa values for organochlorines and herbicides remained below 1 across all age groups, as illustrated by the uniform green gradient indicating no significant acute cumulative risk under the conditions of this study.

Importantly, the overall risk profile shifts completely when examining long-term exposure. As shown in Figure 4b, all chronic cumulative hazard indices (HIc) remained strictly below 1 across all pesticide groups and age categories. This indicates that long-term cumulative exposure to these pesticide mixtures through mango consumption alone is unlikely to cause appreciable non-carcinogenic health effects. This finding is consistent with other cumulative dietary risk assessments, which often report low chronic HI values for pesticide mixtures in fruits and cereals, despite occasional concerns about acute cumulative exposure in high-consumption scenarios or specific age groups. Overall, these findings suggest that acute mixture exposure, particularly to pyrethroids and carbamates, deserves greater attention from a risk management perspective. In contrast, chronic cumulative risks associated with mango consumption appear to remain within acceptable limits under current residue levels and consumption.

## 4. Conclusions

The present study investigated multi-residue pesticide contamination in mango and characterized the associated human health risks. A EN QuEChERS protocol combined with GC-MS/MS was validated for the determination of 44 pesticides and applied to 255 mango samples collected from Phichit, Phitsanulok, and Phetchabun provinces in Thailand.

Of the detected pesticides, the mean concentrations of 22 compounds exceeded the Thai MRLs, and 14 compounds exceeded the Codex MRLs, highlighting regulatory non-compliance in a proportion of the analyzed samples. The acute dietary exposure assessment indicated that carbofuran, lambda-cyhalothrin, and fenpropathrin were the principal contributors to acute health risk, with HQa values greater than 1 across all age groups. At the cumulative level, organophosphate, carbamate, and pyrethroid pesticides also produced HIa values greater than 1 in most age groups, suggesting potential concern under worst-case high-exposure scenarios. In contrast, organochlorine pesticides and atrazine remained below the acute risk threshold. Furthermore, both individual HQc and cumulative HIc values remained below 1 for all pesticide groups and age categories, indicating that long-term exposure to pesticide residues through mango consumption is unlikely to pose appreciable non-carcinogenic health risks.

Overall, these findings indicate that although chronic risks associated with mango consumption appear to be acceptable, certain highly toxic insecticides may pose non-negligible acute health risks under worst-case exposure scenarios. Nevertheless, the findings should be interpreted in light of limitations. First, the samples were collected from only three provinces and may not reflect seasonal and geographical variation across all Thai mango production areas. Second, the analytical scope was limited to the 44 target pesticides included in the GC–MS/MS method, and no untargeted screening was conducted; therefore, pesticide residues outside the target list, as well as unknown or unexpected pesticide-related compounds, could not be assessed. Third, because the dietary risk assessment was based on Thai food-consumption data and body-weight assumptions, the findings are primarily applicable to the Thai population and should not be directly extrapolated to populations in other countries with different dietary consumption patterns. Future studies should include broader geographical and seasonal sampling, expanded targeted analytical coverage, untargeted screening approaches, and country-specific consumption data for major export markets. Continued residue monitoring, implementation of good agricultural practices, and risk communication are essential to reduce pesticide exposure and protect consumers.

## Figures and Tables

**Table 1:** Body weight, large-portion consumption and average daily data of different age groups.

Age Group (Years)	Average Body Weight (kg)	Large-Portion Consumption (P97.5) (kg/Day)	Average Daily Consumption (kg/Day)
3–5.9	17.25	0.255	0.147
6–12.9	33.38	0.510	0.188
13–17.9	53.42	0.255	0.218
18–34.9	63.12	0.510	0.244
35–64.9	63.53	0.510	0.214
>65	55.77	0.510	0.170

Source: Adapted from Thailand Food Consumption Database, National Bureau of Agricultural Commodity and Food Standards (ACFS), Thailand (2016).

**Table 2:** Validation parameters of the QuEChERS–GC–MS/MS method for the determination of pesticide residues in mango matrix.

No.	Compound Name	Mass Transition (*m*/*z*)	Linearity (r^2^)	Limits of Detection (µg/kg)	Limits of Quantitation (µg/kg)	%RSD for 100 ug/kg (*n* = 10)	%Recovery Spike Level (*n* = 3)		
							Low (10 µg/kg)	Medium (100 µg/kg)	High (500 µg/kg)
1	Dichlorvos	185.0 → 93.0	0.9994	1.18	3.59	13.21	99.07	101.99	98.24
2	Methamidophos	141.0 → 95.0	0.9994	12.54	38.01	11.93	116.04	99.13	99.37
3	Mevinphos	127.0 → 109.0	0.9997	8.06	24.42	2.55	116.54	97.66	101.21
4	Acephate	136.0 → 94.0	0.9899	26.40	80.00	9.81	113.26	84.05	97.06
5	Omethoate	156.0 → 79.0	0.9939	24.74	74.96	8.34	113.13	74.04	100.95
6	Monocrotophos	127.1 → 109.0	0.9914	1.02	3.09	12.80	116.62	83.12	98.37
7	Dicrotophos	127.0 → 109.0	0.9954	0.75	2.28	15.22	113.26	78.68	101.18
8	Dimethoate	87.0 → 46.0	0.9994	11.55	35.00	10.08	80.82	94.57	100.35
9	Diazinon	137.1 → 84.0	0.9976	12.07	36.58	1.02	80.45	100.6	98.30
10	Pirimiphos-methyl	232.0 → 125.0	0.9814	7.75	23.49	10.96	115.69	79.56	105.11
11	Fenitrothion	125.0 → 47.0	0.9964	5.05	15.31	8.30	118.22	97.70	102.1
12	Parathion-methyl	125.0 → 47.0	0.9958	8.83	26.76	3.43	115.3	94.27	102.25
13	Malathion	126.9 → 99.0	0.9995	1.21	3.67	2.86	118.01	93.19	99.96
14	Chlorpyrifos	196.9 → 169.0	0.9988	2.47	7.48	1.00	105.08	99.50	98.28
15	Methidathion	144.9 → 85.0	0.9995	1.69	5.12	5.85	114.85	93.68	100.00
16	Prothiofos	266.9 → 239.0	0.9989	0.34	1.02	5.47	112.16	96.40	99.83
17	Profenofos	207.9 → 63.0	0.9971	12.12	36.72	12.86	111.45	88.39	101.68
18	Ethion	231.0 → 129.0	0.9910	1.19	3.60	3.69	114.94	84.07	104.91
19	Triazophos	161.2 → 134.2	0.9990	1.84	5.59	7.65	118.5	90.78	99.91
20	Phosmet	160.0 → 77.1	0.9950	5.88	17.81	3.02	111.54	85.06	102.12
21	Coumaphos	361.9 → 109.0	0.9873	0.85	2.57	7.86	119.78	81.93	106.28
22	Azinphos-ethyl	132.0 → 77.1	0.9934	3.13	9.49	3.77	105.92	83.32	102.56
23	Azinphos-methyl	160.0 → 77.0	0.9915	0.68	2.06	3.93	112.76	84.63	102.99
24	EPN	169.0 → 77.0	0.9981	2.09	6.34	4.57	114.96	86.70	101.00
25	Oxamyl	98.0 → 58.0	0.9948	0.19	0.57	10.01	121.39	97.77	99.81
26	Fenobucarb	121.0 → 77.0	0.9991	0.50	1.50	2.72	78.48	100.71	98.95
27	Carbofuran	161.2 → 134.2	0.9958	14.40	43.65	8.79	119.75	96.05	102.3
28	Pirimicarb	238.0 → 166.2	0.9995	4.75	14.39	3.02	101.82	100.70	98.99
29	Carbaryl	144.1 → 116.1	0.9985	1.98	5.99	13.07	93.28	96.88	99.01
30	Carbosulfan	149.1 → 77.1	0.9979	0.76	2.30	3.71	93.89	98.53	98.49
31	Lambda-cyhalothrin	181.1 → 152.1	0.9953	2.41	7.31	7.33	107.32	85.27	102.11
32	Fenpropatrin	181.0 → 152.0	0.9956	4.79	14.52	10.1	115.87	84.58	102.00
33	Permethrin	183.1 → 153.1	0.9996	7.58	22.98	2.80	112.79	94.13	100.14
34	Cyfluthrin	163.0 → 90.9	0.9986	5.50	16.68	6.43	110.04	86.19	85.21
35	Cypermethrin	163.0 → 91.0	0.9998	7.51	22.75	1.99	119.04	92.27	100.69
36	Fluvalinate-tau	181.0 → 152.0	0.9978	18.42	55.82	1.02	118.37	89.75	101.44
37	Fenvalerate	181.0 → 152.1	0.9964	6.55	19.85	1.01	89.07	86.12	101.68
38	Esfenvalerate	167.0 → 125.1	0.9970	2.91	8.81	1.44	109.12	86.51	101.45
39	Deltamethrin	253.0 → 93.0	0.9907	10.80	32.72	15.58	119.53	99.03	106.06
40	Aldrin	263.0 → 191.0	0.9991	5.12	15.52	2.67	95.53	99.46	98.16
41	Endosulfan	195.0 → 125.0	0.9563	7.70	23.32	3.04	112.03	91.34	106.48
42	DDT-o,p′	235.0 → 165.2	0.9994	14.29	43.29	3.40	102.46	94.85	100.33
43	Amitraz	132.1 → 117.1	0.9996	1.18	3.59	9.45	105.12	94.60	100.24
44	Atrazine	215.0 → 58.0	0.9992	12.54	38.01	7.83	110.27	95.10	100.22

**Table 3:** The detection frequency, residue results of pesticide and the MRLs in mango samples.

No.	Pesticides	No. of Detectable Samples (%)	Mean (µg/kg)	Min–Max (µg/kg)	MRLs (µg/kg)		Exceedance Status
					Thailand	Codex	
**Organophosphate**							
1	Dichlorvos	3 (1.2)	9.6	1.7–17.5	10 *	10 *	-
2	Methamidophos	58 (22.7)	23.5	20.3–38.6	10 *	10 *	Thailand, Codex
3	Mevinphos	1 (0.4)	26.7	26.7–26.7	10 *	NE	Thailand
4	Acephate	84 (32.9)	106.2	80.1–164.5	10 *	10 *	Thailand, Codex
5	Omethoate	62 (24.3)	29.8	24.1–44.0	10 *	NE	Thailand
6	Monocrotophos	34 (13.3)	37.5	26.5–98.9	10 *	NE	Thailand
7	Dicrotophos	54 (21.2)	25.8	22.3–40.7	10 *	10 *	Thailand
8	Dimethoate	24 (9.4)	12	10.3–34.0	1000	1000	-
9	Diazinon	11 (4.3)	12.8	0.9–27.5	500	500	-
10	Pirimiphos-methyl	5 (2.0)	94.3	51.6–174.3	500	500	-
11	Fenitrothion	28 (11.0)	15.7	14.4–27.5	1000	1000	-
12	Parathion-methyl	29 (11.4)	11.6	10.5–25.3	500	500	-
13	Malathion	57 (22.4)	10.5	5.7–41.2	10 *	NE	Thailand
14	Chlorpyrifos	134 (52.5)	3.8	0.1–56.5	10 *	NE	-
15	Methidathion	109 (42.7)	43.2	6.5–204.8	1000	NE	-
16	Prothiofos	11 (4.3)	18.7	2.4–68.7	10 *	NE	Thailand
17	Profenofos	35 (13.7)	19.8	2.4–63.3	10 *	10 *	Thailand, Codex
18	Ethion	29 (11.4)	16.8	7.0–125.7	10 *	NE	Thailand
19	Triazophos	17 (6.7)	19.1	9.3–39.5	2000	2000	-
20	Phosmet	14 (5.5)	21.3	19.8–34.5	10 *	100	Thailand
21	Coumaphos	36 (14.1)	8.4	7.5–34.7	2000	2000	-
22	Azinphos-ethyl	30 (11.8)	23.4	20.4–44.0	10 *	10 *	Thailand, Codex
23	Azinphos-methyl	25 (9.8)	26.3	25.9–29.3	10 *	10 *	Thailand, Codex
24	EPN	34 (13.3)	77	11.4–202.0	3000	3000	-
**Carbamate**							
25	Oxamyl	49 (19.2)	98.3	74.0–263.0	10 *	10 *	Thailand, Codex
26	Fenobucarb	85 (33.3)	93.3	0.3–2907.2	10 *	10 *	Thailand, Codex
27	Carbofuran	96 (37.6)	54.9	11.0–362.7	10 *	NE	Thailand,
28	Pirimicarb	ND	ND	ND	10 *	10 *	-
29	Carbaryl	40 (15.7)	8.3	3.2–68.8	5000	5000	-
30	Carbosulfan	17 (6.7)	24	8.2–43.0	10 *	10 *	Thailand, Codex
**Pyrethroid**							
31	Lambda-cyhalothrin	97 (38.0)	1009.4	18.5–10,272.4	10 *	10 *	Thailand, Codex
32	Fenpropatrin	89 (34.9)	1222.4	34.8–11,439.1	300	300	Thailand, Codex
33	Permethrin	26 (10.2)	50.9	5.6–155.5	10 *	10 *	Thailand, Codex
34	Cyfluthrin	97 (38.0)	40.2	5.7–150.3	10 *	10 *	Thailand, Codex
35	Cypermethrin	73 (28.6)	105.3	35.5–1618.0	500	500	-
36	Fluvalinate-tau	25 (9.8)	25.9	13.8–72.9	200	200	-
37	Fenvalerate	50 (19.6)	17.6	7.3–60.2	10 *	10 *	Thailand, Codex
38	Esfenvalerate	131 (51.4)	31.9	18.5–67.5	200	200	-
39	Deltamethrin	19 (7.5)	31.3	31.3–299.8	200	200	-
**Organochlorine**							
40	Aldrin	78 (30.6)	8.4	0.3–87.3	500	500	-
41	Endosulfan	11 (4.3)	20.1	2.6–71.8	500	500	-
42	DDT-o,p′	18 (7.1)	3	2.3–4.7	10 *	5000	-
43	Amitraz	117 (45.9)	61.4	6.3–156.3	2000	10 *	Codex
**Herbicide**							
44	Atrazine	68 (26.7)	0.6	0.6–0.9	10 *	NE	-

* = default MRLs according to Thai Ministry of Public Health regulations and the Codex Alimentarius standards, which stipulate a default value of 10 µg/kg for active substances for which no commodity-specific MRL has been established. NE = Not established, indicating that no pesticide–commodity-specific MRLs have been established for mango. Note: The pesticide class headings—Organophosphate, Carbamate, Pyrethroid, Organochlorine, and Herbicide—are shown in bold for clarity.

**Table 4:** Acute dietary exposure (ESTI) and hazard quotients (HQa) of individual pesticides via mango consumption across age groups.

No.	Pesticide	Highest Residue (µg/kg)	ARfD (µg/kg BW)	ESTI (µg/kg BW)						HQa (%)					
				3–5.9	6–12.9	13–17.9	18–34.9	35–64.9	>65	3–5.9	6–12.9	13–17.9	18–34.9	35–64.9	>65
	Organophosphate														
1	Dichlorvos	17.488	10	0.259	0.267	0.083	0.141	0.140	0.160	2.585	2.672	0.835	1.413	1.404	1.599
2	Methamidophos	38.648	10	0.571	0.590	0.184	0.312	0.310	0.353	5.713	5.905	1.845	3.123	3.103	3.534
3	Mevinphos	26.675	3	0.394	0.408	0.127	0.216	0.214	0.244	13.144	13.585	4.244	7.184	7.138	8.131
4	Acephate	164.461	100	2.431	2.513	0.785	1.329	1.320	1.504	2.431	2.513	0.785	1.329	1.320	1.504
5	Omethoate	43.996	2	0.650	0.672	0.210	0.355	0.353	0.402	32.519	33.610	10.501	17.774	17.659	20.117
6	Monocrotophos	98.934	2	1.463	1.512	0.472	0.799	0.794	0.905	73.125	75.579	23.613	39.969	39.711	45.236
7	Dicrotophos	40.748	-	-	-	-	-	-	-	-	-	-	-	-	-
8	Dimethoate	33.985	20	0.502	0.519	0.162	0.275	0.273	0.311	2.512	2.596	0.811	1.373	1.364	1.554
9	Diazinon	27.496	30	0.406	0.420	0.131	0.222	0.221	0.251	1.355	1.400	0.438	0.741	0.736	0.838
10	Pirimiphos-methyl	174.296	200	2.577	2.663	0.832	1.408	1.399	1.594	1.288	1.332	0.416	0.704	0.700	0.797
11	Fenitrothion	27.480	40	0.406	0.420	0.131	0.222	0.221	0.251	1.016	1.050	0.328	0.555	0.552	0.628
12	Parathion-methyl	25.320	30	0.374	0.387	0.121	0.205	0.203	0.232	1.248	1.290	0.403	0.682	0.678	0.772
13	Malathion	41.200	2000	0.609	0.629	0.197	0.333	0.331	0.377	0.030	0.031	0.010	0.017	0.017	0.019
14	Chlorpyrifos	56.459	100	0.835	0.863	0.270	0.456	0.453	0.516	0.835	0.863	0.270	0.456	0.453	0.516
15	Methidathion	204.821	10	3.028	3.129	0.978	1.655	1.644	1.873	30.278	31.294	9.777	16.549	16.442	18.730
16	Prothiofos	68.673	10	1.015	1.049	0.328	0.555	0.551	0.628	10.152	10.492	3.278	5.549	5.513	6.280
17	Profenofos	63.335	1000	0.936	0.968	0.302	0.512	0.508	0.579	0.094	0.097	0.030	0.051	0.051	0.058
18	Ethion	125.700	-	-	-	-	-	-	-	-	-	-	-	-	-
19	Triazophos	39.501	1	0.584	0.604	0.189	0.319	0.317	0.361	58.393	60.352	18.856	31.916	31.710	36.122
20	Phosmet	34.525	30	0.510	0.527	0.165	0.279	0.277	0.316	1.701	1.758	0.549	0.930	0.924	1.052
21	Coumaphos	34.671	50	0.513	0.530	0.166	0.280	0.278	0.317	1.025	1.059	0.331	0.560	0.557	0.634
22	Azinphos-methyl	43.972	100	0.650	0.672	0.210	0.355	0.353	0.402	0.650	0.672	0.210	0.355	0.353	0.402
23	Azinphos-ethyl	29.315	-	-	-	-	-	-	-	-	-	-	-	-	-
24	EPN	202.006	100	2.986	3.086	0.964	1.632	1.622	1.847	2.986	3.086	0.964	1.632	1.622	1.847
	Carbamate														
25	Oxamyl	263.022	9	3.888	4.019	1.256	2.125	2.111	2.405	43.202	44.651	13.950	23.613	23.461	26.725
26	Fenobucarb (BPMC)	2907.165	-	-	-	-	-	-	-	-	-	-	-	-	-
27	Carbofuran	362.728	1	5.362	5.542	1.731	2.931	2.912	3.317	536.206	554.197	173.148	293.078	291.187	331.703
28	Pirimicarb	0.096	100	0.001	0.001	0.000	0.001	0.001	0.001	0.001	0.001	0.000	0.001	0.001	0.001
29	Carbaryl	68.787	200	1.017	1.051	0.328	0.556	0.552	0.629	0.508	0.525	0.164	0.278	0.276	0.315
30	Carbosulfan	43.040	-	-	-	-	-	-	-	43.202	44.651	13.950	23.613	23.461	26.725
	Pyrethroid														
31	Lambda-cyhalothrin	10,272.443	20	151.854	156.949	49.035	83.000	82.464	93.938	759.268	784.743	245.177	414.999	412.321	469.692
32	Fenpropatrin	11,439.066	30	169.099	174.773	54.604	92.426	91.829	104.607	563.664	582.577	182.014	308.086	306.098	348.689
33	Permethrin	155.527	50	2.299	2.376	0.742	1.257	1.249	1.422	4.598	4.752	1.485	2.513	2.497	2.844
34	Cyfluthrin	150.259	40	2.221	2.296	0.717	1.214	1.206	1.374	5.553	5.739	1.793	3.035	3.016	3.435
35	Cypermethrin	1618.029	40	23.919	24.721	7.724	13.073	12.989	14.796	59.797	61.803	19.309	32.684	32.473	36.991
36	Fluvalinate-tau	72.917	500	1.078	1.114	0.348	0.589	0.585	0.667	0.216	0.223	0.070	0.118	0.117	0.133
37	Fenvalerate	60.170	200	0.889	0.919	0.287	0.486	0.483	0.550	0.445	0.460	0.144	0.243	0.242	0.275
38	Esfenvalerate	67.547	20	0.999	1.032	0.322	0.546	0.542	0.618	4.993	5.160	1.612	2.729	2.711	3.088
39	Deltamethrin	299.849	50	4.433	4.581	1.431	2.423	2.407	2.742	8.865	9.163	2.863	4.845	4.814	5.484
	Organochlorine														
40	Aldrin	87.335	-	-	-	-	-	-	-	-	-	-	-	-	-
41	Endosulfan	71.756	20	1.061	1.096	0.343	0.580	0.576	0.656	5.304	5.482	1.713	2.899	2.880	3.281
42	DDT-o,p′	4.746	-	-	-	-	-	-	-	-	-	-	-	-	-
43	Amitraz	156.279	10	2.310	2.388	0.746	1.263	1.255	1.429	23.102	23.877	7.460	12.627	12.546	14.291
	Herbicide														
44	Atrazine	0.918	10	0.014	0.014	0.004	0.007	0.007	0.008	0.136	0.140	0.044	0.074	0.074	0.084

Source of ADI and ARfD: JMPR (Joint FAO/WHO Meeting on Pesticide Residues) [12,14].

**Table 5:** Chronic dietary exposure (EDI) and hazard quotients (HQc) of individual pesticides via mango consumption across age groups.

No.	Pesticide	Mean Residue (µg/kg)	ADI (µg/kg BW)	EDI (µg/kg BW)						HQc (%)					
				3–5.9	6–12.9	13–17.9	18–34.9	35–64.9	>65	3–5.9	6–12.9	13–17.9	18–34.9	35–64.9	>65
	Organophosphate														
1	Dichlorvos	0.075	4	0.001	0.000	0.000	0.000	0.000	0.000	0.016	0.011	0.008	0.007	0.006	0.006
2	Methamidophos	5.343	4	0.045	0.030	0.022	0.021	0.018	0.016	1.135	0.752	0.546	0.516	0.451	0.407
3	Mevinphos	0.105	0.8	0.001	0.001	0.000	0.000	0.000	0.000	0.111	0.074	0.053	0.050	0.044	0.040
4	Acephate	34.994	30	0.297	0.197	0.143	0.135	0.118	0.106	0.991	0.657	0.477	0.450	0.394	0.355
5	Omethoate	7.251	4	0.062	0.041	0.030	0.028	0.024	0.022	1.540	1.021	0.741	0.700	0.612	0.552
6	Monocrotophos	5.003	0.6	0.042	0.028	0.020	0.019	0.017	0.015	7.083	4.696	3.408	3.219	2.813	2.537
7	Dicrotophos	5.470	0.1	0.046	0.031	0.022	0.021	0.018	0.017	46.465	30.810	22.359	21.119	18.453	16.647
8	Dimethoate	1.130	1	0.010	0.006	0.005	0.004	0.004	0.003	0.960	0.637	0.462	0.436	0.381	0.344
9	Diazinon	0.551	3	0.005	0.003	0.002	0.002	0.002	0.002	0.156	0.103	0.075	0.071	0.062	0.056
10	Pirimiphos-methyl	1.850	30	0.016	0.010	0.008	0.007	0.006	0.006	0.052	0.035	0.025	0.024	0.021	0.019
11	Fenitrothion	1.782	6	0.015	0.010	0.007	0.007	0.006	0.005	0.252	0.167	0.121	0.115	0.100	0.090
12	Parathion-methyl	1.323	3	0.011	0.007	0.005	0.005	0.004	0.004	0.375	0.248	0.180	0.170	0.149	0.134
13	Malathion	2.353	300	0.020	0.013	0.010	0.009	0.008	0.007	0.007	0.004	0.003	0.003	0.003	0.002
14	Chlorpyrifos	1.881	10	0.016	0.011	0.008	0.007	0.006	0.006	0.160	0.106	0.077	0.073	0.063	0.057
15	Methidathion	18.457	2	0.157	0.104	0.075	0.071	0.062	0.056	7.839	5.198	3.772	3.563	3.113	2.808
16	Prothiofos	0.809	0.1	0.007	0.005	0.003	0.003	0.003	0.002	6.869	4.555	3.305	3.122	2.728	2.461
17	Profenofos	2.721	30	0.023	0.015	0.011	0.011	0.009	0.008	0.077	0.051	0.037	0.035	0.031	0.028
18	Ethion	1.909	2	0.016	0.011	0.008	0.007	0.006	0.006	0.811	0.538	0.390	0.368	0.322	0.290
19	Triazophos	1.272	1	0.011	0.007	0.005	0.005	0.004	0.004	1.080	0.716	0.520	0.491	0.429	0.387
20	Phosmet	1.170	6	0.010	0.007	0.005	0.005	0.004	0.004	0.166	0.110	0.080	0.075	0.066	0.059
21	Coumaphos	1.180	0.5	0.010	0.007	0.005	0.005	0.004	0.004	2.004	1.329	0.965	0.911	0.796	0.718
22	Azinphos-methyl	2.754	30	0.023	0.016	0.011	0.011	0.009	0.008	0.078	0.052	0.038	0.035	0.031	0.028
23	Azinphos-ethyl	2.577	30	0.022	0.015	0.011	0.010	0.009	0.008	0.073	0.048	0.035	0.033	0.029	0.026
24	EPN	10.261	1.4	0.087	0.058	0.042	0.040	0.035	0.031	6.226	4.128	2.996	2.830	2.473	2.230
	Carbamate														
25	Oxamyl	18.884	9	0.160	0.106	0.077	0.073	0.064	0.057	1.782	1.182	0.858	0.810	0.708	0.639
26	Fenobucarb (BPMC)	31.110	5	0.264	0.175	0.127	0.120	0.105	0.095	5.285	3.505	2.543	2.402	2.099	1.894
27	Carbofuran	20.655	1	0.175	0.116	0.084	0.080	0.070	0.063	17.546	11.634	8.443	7.974	6.968	6.286
28	Pirimicarb	0.000	20	0.000	0.000	0.000	0.000	0.000	0.000	0.000	0.000	0.000	0.000	0.000	0.000
29	Carbaryl	1.299	8	0.011	0.007	0.005	0.005	0.004	0.004	0.138	0.091	0.066	0.063	0.055	0.049
30	Carbosulfan	1.602	6	0.014	0.009	0.007	0.006	0.005	0.005	0.227	0.150	0.109	0.103	0.090	0.081
	Pyrethroid														
31	Lambda-cyhalothrin	383.955	20	3.262	2.163	1.569	1.482	1.295	1.168	16.308	10.813	7.847	7.412	6.476	5.842
32	Fenpropatrin	426.641	30	3.624	2.403	1.744	1.647	1.439	1.298	12.080	8.010	5.813	5.491	4.798	4.328
33	Permethrin	5.193	50	0.044	0.029	0.021	0.020	0.018	0.016	0.088	0.059	0.042	0.040	0.035	0.032
34	Cyfluthrin	15.286	40	0.130	0.086	0.062	0.059	0.052	0.047	0.325	0.215	0.156	0.148	0.129	0.116
35	Cypermethrin	30.141	20	0.256	0.170	0.123	0.116	0.102	0.092	1.280	0.849	0.616	0.582	0.508	0.459
36	Fluvalinate-tau	2.542	5	0.022	0.014	0.010	0.010	0.009	0.008	0.432	0.286	0.208	0.196	0.172	0.155
37	Fenvalerate	3.481	20	0.030	0.020	0.014	0.013	0.012	0.011	0.148	0.098	0.071	0.067	0.059	0.053
38	Esfenvalerate	16.397	20	0.139	0.092	0.067	0.063	0.055	0.050	0.696	0.462	0.335	0.317	0.277	0.249
39	Deltamethrin	2.336	10	0.020	0.013	0.010	0.009	0.008	0.007	0.198	0.132	0.095	0.090	0.079	0.071
	Organochlorine														
40	Aldrin	2.571	0.1	0.022	0.014	0.011	0.010	0.009	0.008	21.837	14.479	10.508	9.925	8.672	7.823
41	Endosulfan	0.867	6	0.007	0.005	0.004	0.003	0.003	0.003	0.123	0.081	0.059	0.056	0.049	0.044
42	DDT-o,p′	0.209	2	0.002	0.001	0.001	0.001	0.001	0.001	0.089	0.059	0.043	0.040	0.035	0.032
43	Amitraz	28.153	10	0.239	0.159	0.115	0.109	0.095	0.086	2.391	1.586	1.151	1.087	0.950	0.857
	Herbicide														
44	Atrazine	0.173	20	0.001	0.001	0.001	0.001	0.001	0.001	0.007	0.005	0.004	0.003	0.003	0.003

Source of ADI and ARfD: JMPR (Joint FAO/WHO Meeting on Pesticide Residues) [12,14].

## Data Availability

The original contributions presented in this study are included in the article. Further inquiries can be directed to the corresponding authors.
